# Prevalence of transfusion-transmitted Chagas disease among multitransfused patients in Brazil

**DOI:** 10.1186/1471-2334-8-5

**Published:** 2008-01-16

**Authors:** Erich V De Paula, Neiva SL Goncales, Serge Xueref, Marcelo Addas-Carvalho, Simone CO Gilli, Rodrigo N Angerami, Fernando L Goncales

**Affiliations:** 1 Hematology and Hemotherapy Center, State University of Campinas, Campinas – SP, Brazil; 2 Pan American Health Organization, Regional Office for the World Health Organization, Washington, D.C., USA; 3 School of Medical Sciences, State University of Campinas, Campinas – SP, Brazil

## Abstract

**Background:**

Blood transfusion has always been an important route for Chagas Disease (CD) transmission. The high prevalence of CD in Latin America and its lifelong asymptomatic clinical picture pose a threat for the safety of the blood supply. The outcome of measures designed to improve transfusion safety can be assessed by evaluating the prevalence of CD among multitransfused patients

**Methods:**

In order to assess the impact of CD control measures on the safety of the blood supply, an observational cross-sectional study was designed to determine the prevalence of CD in 351 highly transfused patients, in which vectorial transmission was excluded. This study compared patients that received transfusion products before (n = 230) and after (n = 121) 1997, when measures to control transfusion-transmitted CD were fully implemented in Brazil.

**Results:**

The study group consisted of 351 patients exposed to high numbers of blood products during their lifetime (median number of units transfused = 51, range 10–2086). A higher prevalence of transfusion-transmitted CD (1.30%) was observed among multitransfused patients that received their first transfusion before 1997, compared with no cases of transfusion-transmitted CD among multitransfused patients transfused after that year. The magnitude of the exposure to blood products was similar among both groups (mean number of units transfused per year of exposure = 25.00 ± 26.46 and 23.99 ± 30.58 respectively; P = 0.75, Mann-Whitney test).

**Conclusion:**

Multiple initiatives aimed to control vector and parental transmission of CD can significantly decrease transfusion-transmitted CD in Brazil. Our data suggest that mandatory donor screening for CD represents the most important measure to interrupt transmission of CD by blood transfusions.

## Background

Chagas disease (CD), caused by the protozoan *Trypanosoma cruzi *is considered the most important parasitic disease of the Americas in terms of socioeconomic impact [1]. Despite major improvements in the control of vectorial transmission in endemic areas, CD still infects nearly 10 million people [2], and transfusions have always been an important route of transmission [3]. Furthermore, since most patients with CD are asymptomatic and unaware of their condition, these potential blood donors represent a serious threat to the safety of the blood supply of non-endemic areas [4].

Since 1988, in Brazil as in many Latin American countries, it is mandatory to screen blood donations for anti-*T. cruzi *antibodies, and since 1997, 100% serological coverage was reached in blood banks. [5]. Concomitantly, the control of vectorial transmission has significantly reduced the prevalence of CD among blood donors [6].

One of the strategies used to assess blood supply safety is to evaluate the prevalence of transfusion-transmitted infections among multitransfused patients [7]. In contrast to numerous studies on prevalence of to *T. cruzi *infection among blood donors, only few reports have described its prevalence among multitransfused patients, all of which were published before donated blood started to be regularly screened [8-10]. Therefore, the aim of this study was (1) to assess the prevalence of CD among multitransfused patients in Brazil, (2) to describe the changes in the prevalence over time, and (3) to describe the epidemiological characteristics of transfusion-transmitted CD, with emphasis on transfusion-related risk factors.

## Methods

Three hundred and fifty-one multitransfused patients were recruited from three health public institutions located in the city of Campinas, Sao Paulo state, Brazil. The metropolitan region of Campinas, with 3.2 million inhabitants, is located in a non-endemic area of CD, in the most densely populated region of Brazil. Informed consent was obtained from all patients or legal guardian, and the study was approved by the local Institution Review Board. Patients were considered multitransfused if they had been transfused with at least 10 units of blood products or blood derivatives divided between at least two occasions, with the last occasion being at least 15 days apart from recruitment date. One unit of blood product was defined as 1 unit of whole blood, 1 unit of packed red blood cells, 1 unit of a platelet concentrate or platelet apheresis, 1 unit of plasma, 1 unit of cryoprecipitate (Cryo) or 1 vial of lyophilized coagulation factor concentrate. Patients who were aware of a positive test for CD before the first transfusion event were excluded from the study. Clinical and epidemiological data were obtained through a standard interview and from the patient's medical records. This interview was performed by one member of the team using a structured questionnaire after consent was obtained. The questionnaire covered clinical and epidemiological details of the transfusion history of the patients such as: diagnosis, type and number of blood products used, date of first transfusion event and number of facilities in which the patient was transfused. The questionnaire also included questions about alternative routes for transmission of common blood-borne pathogens such as intravenous drug use and previous history of invasive medical interventions and/or alternative medical interventions. In order to exclude other routes of CD transmission in the study population, patients were questioned about living in CD-endemic areas, in dwellings with mud/mud-brick walls or known to be infested with triatomine bugs. Patients were also questioned about relatives or other household contacts with confirmed diagnosis of CD.

Each calendar-year during which the patient received any number of transfusions was recorded as one year of exposure to transfusions. Donor exposure was estimated assuming 1 unit:1 donor for all blood products, except lyophilized factor concentrates that were not included in this estimation, because of the assumption that *T. cruzi *lose viability during the production of lyophilized clotting factor concentrates. In fact, there are no reports of transmission of CD by these types of blood products.

Blood samples were drawn at the day of recruitment. Serum samples were tested in duplicate using an enzyme immunoassay (Hemobio Chagas, Embrabio, São Paulo, SP, Brazil), according to manufacturer's instructions. The results of anti-*T. cruzi *test were expressed as the optical densities of the samples (S) divided by the cut-off value (C). S/C values ≤ 0.8 were considered seronegative, S/C values between 0.8 and 1.2 were considered indeterminate and S/C values ≥ 1.2 were considered seropositive. Positive results were confirmed in a second sample, specifically collected for to confirm these results, by immune-fluorescence assay (Imuno Cruzi and Fluoline H, Biolab-Meriéux, RJ, Brazil). For the purpose of this study, samples were considered true positive if they were seropositive in both tests.

The observed prevalence of CD was then calculated. To estimate the impact on the safety of the blood supply of the implementation of 100% coverage of serological screening of CD among blood donors, January 1^st ^1997 was selected to define the experimental groups. The mean number of units transfused per year of exposure was compared using the Mann-Whitney test and a P < 0.05 was considered significant.

## Results

Clinical characteristics and details of the transfusion history of the 351 eligible patients from the study are shown in table 1. Three cases of transfusion-transmitted CD were identified. None of of them presented additional risk factors for CD that might suggest an alternative route of transmission. In fact, none of the patients in our study presented additional epidemiological risk factors for CD other than a history of blood transfusions. These 3 cases represented a prevalence rate of CD of 1.30% among patients who were first transfused before 1997, whereas no cases of transfusion-transmitted CD were observed among patients that were transfused only after 1997.

**Table 1:** Characteristics of patients included in the study (n = 351)

**Age **(Mean ± SD)	36.0 ± 19.8
**Sex distribution **(%)	
Male	199 (56.7%)
Female	152 (43.3%)
**Diagnosis responsible for multiple transfusions **– number of patients (%)	
Haemophilia	28 (8.0%)
Haemoglobinopathies	97 (27.6%)
Onco-haematological disease	184 (52.4%)
Acute blood loss	19 (5.4%)
Haemodyalisis	23 (6.6%)
**Units transfused per patient **(Median/Range)	51 (10–2086)
**Years exposed to transfusions **(Median/Range)	3 (1–52)
**1^st ^transfusion before 1997 **(%)	230 (65.5%)

SD- standard deviation. Table 1 presents characteristics of the exposure to multiple transfusions in the study population, such as diagnosis responsible for transfusions and the magnitude of exposure to blood products in their lifetime. This magnitude is expressed both in terms of the number of blood products (units) and calendar-years during which the patients received blood transfusions. In addition, the table also shows the proportion of patients transfused before 1997, the year when multiple initiatives aimed to control transfusion-transmitted CD were fully implemented in Brazil.

*T. cruzi *infected-patients included a hemophiliac who had been transfused with whole blood, packed red blood cells (PRBC), cryo and lyophilized factor concentrate since 1980 and two patients with hemoglobin diseases. The first one is still under regular transfusion program with PRBC (since 1975), and the other (Sβ Thal) was transfused only two times (1971 and 1996). The patient with hemophilia had only received factor concentrate after 1997 (table 2). The mean number of units transfused per year of exposure for these patients was 25.00 ± 26.46, compared to 23.99 ± 30.58 among seronegative patients (P = 0.75; Mann-Whitney test).

**Table 2:** Characteristics of patients seropositive for Chagas disease.

**Diagnosis**	**Year of 1^st ^Transfusion**	**Units transfused (before/after 1997)**	**Other risk factors for Chagas Disease**	Estimated donor exposure *
Hemophilia	1980	72/262	None	50
Thalassemia	1971	1297/253	None	1550
S/β Thal	1975	10/0	None	10

* Donor exposure was estimated assuming 1 unit blood product:1 donor for all blood products, except lyophilized factor concentrates that were not included in this estimation.

## Discussion

In this study we evaluated a population of 351 patients exposed to an extremely high number of blood transfusions for multiple reasons. The possibility of vectorial or other forms of transmission of CD among seropositive patients was discarded by the exclusion of patients with a previous diagnosis of CD, and by an extensive interview covering other known epidemiological risk factors for CD. The laboratory strategy used to assess the prevalence of CD was in agreement with current regulations established by the Brazilian National Health Surveillance Agency for the screening of blood donors for CD [11], and all positive samples were confirmed by an indirect immunofluorescence test in a different sample. To our knowledge, this is the first study to report the prevalence of CD among multitransfused patients after the implementation of serological screening. Given the substantial social and economical regional differences observed in Brazil, our results cannot be extended to the whole country, where prevalence rates and transmission patterns of CD are expected to differ. However, the fact that this study was conducted in an area without vectorial transmission strengthens the association between our results and changes in the risk for transfusion-transmitted CD.

The risk of transfusion-transmitted CD depends on five factors: the prevalence of infection among blood donors, the proportion of blood units actually screened in an area, characteristics of the tests used, the risk of infection per unit of blood transfused and the number of blood units transfused [12]. Assuming that the risk of infection per unit of blood transfused has not substantially changed over time, and considering that the magnitude of exposure to blood products in our study population was high, both in terms of number of units (median 51, range 10 – 2086) and of length in years (median 3, range 1 – 52), we can infer that the chance of acquiring CD among our patients was largely dependent on the first 3 factors.

The main finding of our study was a clear difference in the prevalence rate of CD between patients transfused before and after 1997, with no new cases of transfusion-transmitted CD in the latter group. This difference was observed despite a similar number of blood products transfused per year of exposure in both groups (25.00 ± 26.46 and 23.99 ± 30.58 units in seropositive and seronegative patients respectively; P = 0.75). Improvements in coverage and quality of screening tests for CD are the most important determinant of this change. Indeed, the proportion of blood services performing at least one serological test for CD increased from 69% in 1988 to 95.3% in 1990 in the state of Sao Paulo [13], reaching 100% in 1997. Concomitantly, the sensitivity of screening tests, as well as alternative screening strategies using combinations of methods, further decreased the risk of *T. cruzi *positive blood units of entering in the blood supply [14]. Finally, the decreasing prevalence of CD among blood donors during the last decades, from a median above 2% reported in the 70's to values in the range of 0.6–0.8% during the last 10 years, are also a determinant of the observed results. In a study with only first-time blood donors in the city of Sao Paulo, the prevalence of CD was still decreasing at a significant rate during the late 90's, from 0.47% in 1995 to 0.34% in 2001, with cumulative rates of 0.39% and 0.48% before and after 1997 respectively [6].

As the prevalence of transfusion-transmitted diseases among multitransfused patients is a good indicator of the safety of the blood supply, our results confirm the assumption that after 1997 blood transfusions no longer represented a significant route for the transmission of CD in the most densely populated area of Brazil.

## Conclusion

In conclusion, the prevalence of CD among multitransfused patients declined significantly as a result of the implementation of multiple interventions aimed to reduce the transmission of CD in Brazil. Permanent vigilance is essential to identify points that could be further improved to minimize the risk of transfusion-transmitted CD such as the accuracy of screening tests.

## Competing interests

The author(s) declare that they have no competing interests.

## Authors' contributions

EVDP participated in the design of the study, coordinated the sample collection, performed interviews and drafted the manuscript. NSLG participated in the design of the study, coordinated the laboratory analyses and helped to draft the manuscript. SX participated in the design and coordination of the study. MA participated in the design of the study and performed the statistical analysis. SCOG participated in the design of the study and performed interviews. RNA participated in the design of the study and coordinated the database. FLG participated in the design and coordination of the study, and helped to draft the manuscript. All authors have read and approved the final manuscript.

## Pre-publication history

The pre-publication history for this paper can be accessed here:


